# A principled link between object naming and representation is available to infants by seven months of age

**DOI:** 10.1038/s41598-023-41538-y

**Published:** 2023-08-31

**Authors:** Alexander LaTourrette, Dana Michelle Chan, Sandra R. Waxman

**Affiliations:** 1 Department of Psychology, University of Pennsylvania, Philadelphia, PA USA; 2 Department of Psychology, Northwestern University, 2029 Sheridan Road, Evanston, IL 60208 USA

**Keywords:** Psychology, Human behaviour, Learning and memory

## Abstract

By their first birthdays, infants represent objects flexibly as a function of not only *whether* but *how* the objects are named. Applying the same name to a set of different objects from the same category supports object categorization, with infants encoding commonalities among objects at the expense of individuating details. In contrast, applying a distinct name to each object supports individuation, with infants encoding distinct features at the expense of categorical information. Here, we consider the development of this nuanced link between naming and representation in infants’ first year. Infants at 12 months (Study 1; N = 55) and 7 months (Study 2; N = 96) participated in an online recognition memory task. All infants saw the same objects, but their recognition of these objects at test varied as a function of how they had been named. At both ages, infants successfully recognized objects that had been named with distinct labels but failed to recognize these objects when they had all been named with the same, consistent label. This new evidence demonstrates that a principled link between object naming and representation is available by 7 months, early enough to support infants as they begin mapping words to meaning.

Human cognition is remarkable for its representational flexibility. We readily represent any given object (e.g., the family pet) as either a member of an object category (e.g., a cat, a mammal) or as a unique individual (e.g., that particular cat, “Sammi”), and we switch effortlessly among these representations. This representational flexibility is supported by language: how an object is *named*—either as an individual or a member of a category—is instrumental to how we *mentally represent* it. This, in turn, has powerful downstream consequences for learning and reasoning about that object^1–7^.

This representational flexibility is supported by language even in infancy, well before infants produce their first words. The most robust evidence for this effect comes from infants’ performance in object categorization tasks. For infants as young as 4 months of age, listening to language facilitates object categorization in a way that other, carefully matched acoustic signals do not^8, 9^. If infants view a series of individuals from the same object category (e.g., several dinosaurs) and each is introduced in conjunction with language (e.g., “Look at the *dax*!”), then infants successfully identify the overarching category at test, looking longer to an individual from a novel category (i.e., a fish) than to a new member of the now-familiar category (i.e., a new dinosaur). In contrast, if the very same individual category members are paired consistently with other acoustic signals (e.g., backward speech, sine-wave tone sequences), infants fail to form the overarching category, looking equally to the novel-category and familiar-category exemplars. Moreover, by 12 months, infants are sensitive not only to *whether*, but *how* objects are named. Providing the same, consistently applied name to a set of individual objects facilitates categorization, but providing a distinct name for each (“Look at the *dax*! Look at the *blicket*!”) does not^10–12^: instead, it promotes object individuation^13–16^.

What mechanism, then, underlies this nuanced effect of naming on infants’ object representations? LaTourrette and Waxman^17^ proposed that naming affects infants’ representation and encoding of objects: while consistent names highlight commonalities among objects, distinct names highlight differences. To test this, they developed a new recognition memory task (Fig. 1). The initial learning phase retained the design of the categorization task. All infants viewed a series of distinct individuals from the same overarching category (animals); these were introduced with either the same, consistently applied label (Consistent Name condition) or with their own unique label (Distinct Names condition). The test phase assessed infants’ memory for each individual seen during Learning. To provide the strongest test of labels’ effect on memory, these individuals appeared in the reverse order at test (see Fig. 1). On four separate test trials, each of these individuals, starting with the one most recently seen at the end of learning, was presented alongside a new animal that infants had never seen. If infants recognize the object they had seen during learning and can distinguish it from the new one, they should prefer looking at the new object. However, if infants fail to recognize the previously seen individual, they should show no preference.

**Figure 1:**
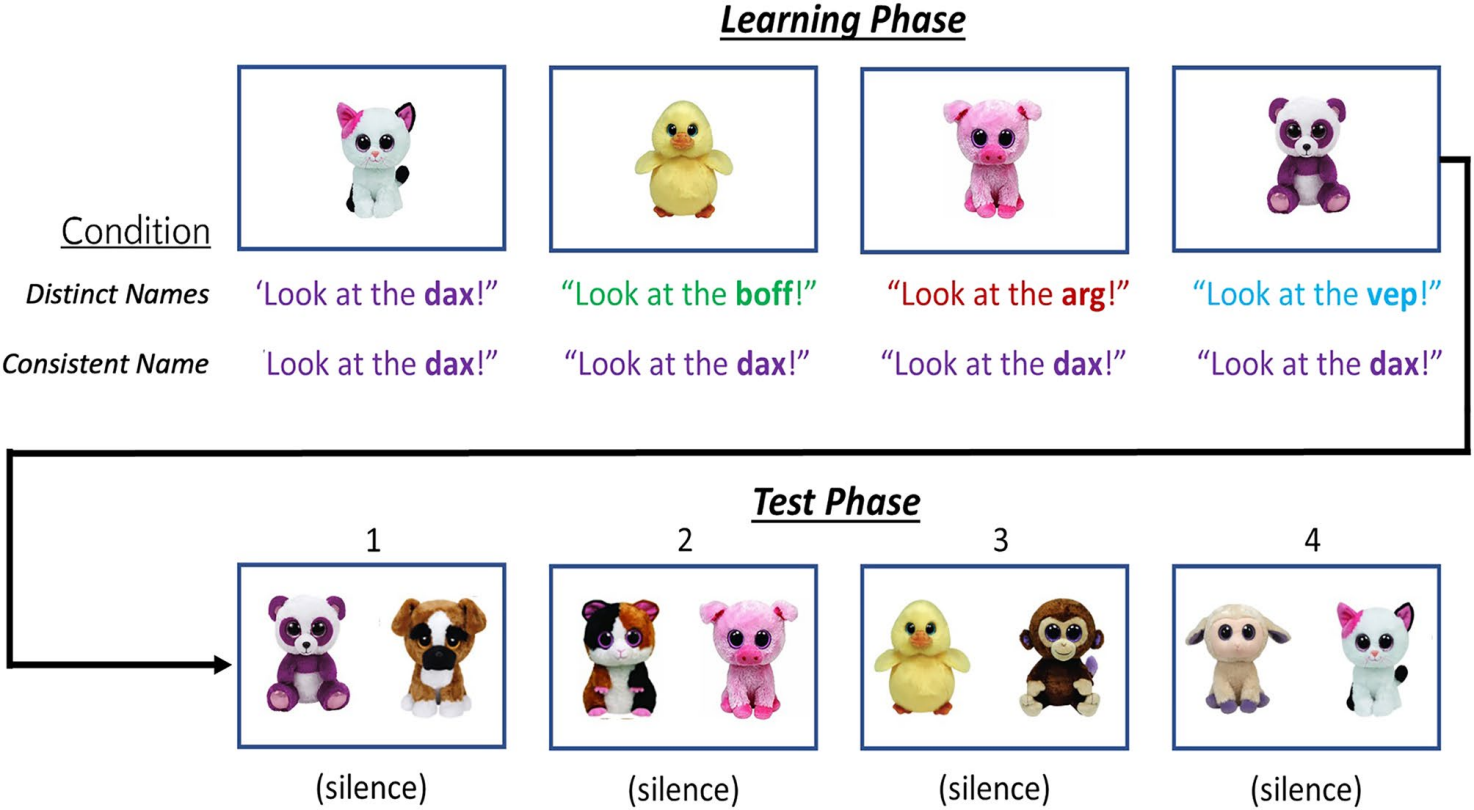
Studies 1 and 2 procedure and representative stimuli. In the Learning Phase, all infants saw images of four stuffed animals, presented one at a time in random order and in conjunction with a novel name. What varied was whether each was labeled with its own distinct name (Distinct Names condition) or with the same consistently applied name (Consistent Name condition). In both conditions, the naming phrase began ~ 150 ms after the image appeared, and again 10 s later. In the Test Phase, all infants saw four test trials, each featuring two images: each animal from the Learning Phase was presented in the reverse order, side-by-side with a novel animal that infants had not seen during Learning. This provided infants with the best chance to recognize the final exemplars seen during Learning, which were predicted to show the greatest effect of labeling. All trials were 20 s in duration.

As predicted, 12-month-old infants’ recognition memory depended on how the individuals had been named. More specifically, on Test trials 1 and 2, which tested memory for the last two objects seen during learning, infants’ performance varied as a function of condition: infants in the Distinct Names condition recognized each individual object, but those in the Consistent Name condition did not—despite having seen them only seconds earlier. Notably, this outcome was specific to naming. Infants in a control condition in which each individual was paired with a consistent sine-wave tone sequence performed differently: they recognized only the most recently seen individual (presented on Test trial 1), exhibiting a classic recency effect. Performance on Test trials 3 and 4, which featured the exemplars seen on the first two Learning trials, did not differ across conditions. This is consistent with the proposal that over the course of Learning, as the same, consistent name was applied to all individuals, infants increasingly focused on category-wide commonalities at the cost of encoding individual-level detail^17–19^, but when distinct names were applied to each individual, this highlighted their unique features—enabling infants to represent them as individuals but at the cost of representing their shared attributes^12, 20^. Thus, even a single naming episode can have a lasting impact, influencing how infants encode that object, represent it in memory, and remember it later.

But how early is this principled link between object naming and object representation available? Perhaps this link must be built upon the bedrock of a rudimentary lexicon. On this view, infants must first acquire a lexicon that includes different words that can be applied to the same individual (e.g., “animal,” “cat,” and “Sammi”); then, they use this lexicon as a foundation for linking the way an object is named to the way they represent it. Alternatively, perhaps infants begin the task of word learning with an expectation that lexical distinctions map onto conceptual distinctions (e.g., with separate words for category-level and individual-level representations)^21–23^. On this view, a link between how objects are named and how they are represented may be available to infants as they first build their lexicons.

To adjudicate between these alternatives, we focus on 7-month-old infants because this is the developmental period at which infants are just beginning to learn the names of a few, highly frequent words ^24^. Certainly, infants’ attentional and memory resources are more limited at 7 than 12 months^25–29^. Nevertheless, 7-month-olds have also mastered several of the requisite skills for the establishment of a link between object naming and object representation. For instance, 7-month-olds successfully segment words from the continuous speech stream^30, 31^, remember objects they have recently seen^25, 26^, and are able to represent objects both as distinct individuals^32, 33^ and as category members^34–38^. In addition, longitudinal training designs suggest that 7-month-old infants may be sensitive to the distinct representational consequences of naming objects with the same versus different names. In multiple studies, Scott and her colleagues^20, 39, 40^ recruited 6-month-old infants to participate in a 3-month training regimen. All infants viewed the same set of images (e.g., monkey faces) presented in a picture book. What varied was whether, over the training period, each image was paired with a unique name or a consistently applied name. At 9 months, the effect of this naming manipulation was apparent. Infants who had heard a distinct name for each training object successfully distinguished these from new members of the same category. In contrast, infants who had heard the same name applied consistently to all training objects failed to distinguish them from new members^20, 39, 40^.

This outcome is impressive but leaves open a key developmental question: is young infants’ sensitivity to how objects are named available only after months of prolonged training, or is this sensitivity present from the moment of labeling? To address this question, we adopted LaTourrette and Waxman’s^17^ paradigm. However, restrictions imposed by the COVID-19 pandemic required that we use an online platform (Lookit)^41^. Because results obtained in laboratories do not always converge with those obtained from online platforms^41–44^, our first goal was to perform a conceptual replication of LaTourrette and Waxman’s^17^ in-lab finding in an online platform (Study 1). As in the original study, 12-month-old infants viewed four different objects, paired with either a consistent name for all objects or a distinct name for each object. Infants were then tested on their ability to discriminate these objects from new category members. This replication provided the foundation for assessing 7-month-old infants in the same paradigm (Study 2). We predicted that despite 7-month-old infants’ more limited attentional and memory resources, their representation and memory for objects would nevertheless be shaped by how the objects were named. Seven-month-old infants’ recognition of individual objects should be stronger if each object had previously been named with a distinct label than with the same, consistently applied label.

In both studies, we adopted a conservative analytic approach in order to align the current results with those reported in the prior in-lab study. Based on that prior study, we predicted that the effects of naming would be evident only within the first two Test trials (corresponding to the last two Learning trials). Nevertheless, we assessed infants’ performance for each object seen during Learning, first reporting the results combined across all four Test trials, and next reporting trial-by-trial analyses to assess the key trial-based predictions outlined below.

## Results

### Study 1: 12 months

Twelve-month-old infants participated in an object recognition memory task identical to LaTourrette and Waxman^17^ but implemented online with the testing platform Lookit^41^ (Fig. 1). To assess the viability of the online testing platform for the questions at hand, we adopted a two-pronged analytic approach, focusing first on infants' performance in the online platform itself and then conducting a direct comparison of results in the online and in-lab platforms^17^.

### Predictions

Following LaTourrette and Waxman^17^, we predicted the effect of naming on infants’ object representation and encoding would emerge gradually over the course of the Learning Phase. On Learning Trial 1, all infants are treated identically (see Fig. 1), so we expected no differences in encoding on this trial. In line with the prior study results, we also expected no encoding differences on Learning Trial 2: infants in both conditions should compare this new object with their representation of the first in order to detect either commonalities or distinctions between them. By Learning Trials 3 and 4, however, we expected that infants would encode the objects differently as a function of their naming condition. Specifically, we predicted that infants in the Distinct Names, but not the Consistent Name, condition would focus on the distinctions among these individuals and as a result, would recognize these objects at Test. Infants should therefore prefer looking at the novel object on Test Trials 1 and 2 (corresponding to Learning Trials 4 and 3, respectively: see Fig. 1).

### Learning phase

There was no difference between conditions in the time that infants spent looking to the objects during Learning, *M*_Distinct_ = 54.40 s (SD = 11.26 s), *M*_Consistent_ = 55.28 s (SD = 10.40 s); *t*(53) = 0.30, *p* = 0.8). Nor was there any difference in looking time during Learning in the present study compared to LaTourrette and Waxman’s^17^ in-lab investigation, *t*(102) = 1.21, *p* = 0.23. Together, these analyses provide assurances that any condition differences at Test, should they emerge, cannot be attributed to differences in infants’ visual attention to the individuals during Learning.

### Test phase

We predicted that infants in the Distinct Names condition would have encoded the unique features of the objects seen during Learning and should therefore recognize these objects at Test, resulting in a preference for looking to the novel object, particularly on the first two Test trials^45, 46^. In contrast, if infants in the Consistent Name condition encoded the commonalities among the Learning objects rather than their unique features, then they should fail to discriminate these previously seen objects from new category members, resulting in them looking equally to the two test objects.

We first constructed a linear mixed effects model predicting infants’ preference for the novel object with sum-coded fixed effects for Condition, Test Trial, and their interaction, a random effect of participant, and trial-by-participant random slopes. This model yielded non-significant effects of Condition (β = 0.039, SE = 0.022, *p* = 0.076), Test Trial (﻿β = 0.035, SE = 0.022, *p* = 0.1), and the Condition × Test Trial interaction (﻿β = 0.041, SE = 0.044, *p* = 0.3). Notably, the difference between conditions was in the same direction as that reported in LaTourrette and Waxman^17^, with infants in the Distinct Names condition showing stronger novelty preferences those in the Consistent Name condition. However, in the online platform, this difference fell short of statistical significance. This is consistent with previous reports of greater variability and smaller effect sizes in results from online platforms than in the laboratory ^41, 56^.

**Figure 2:**
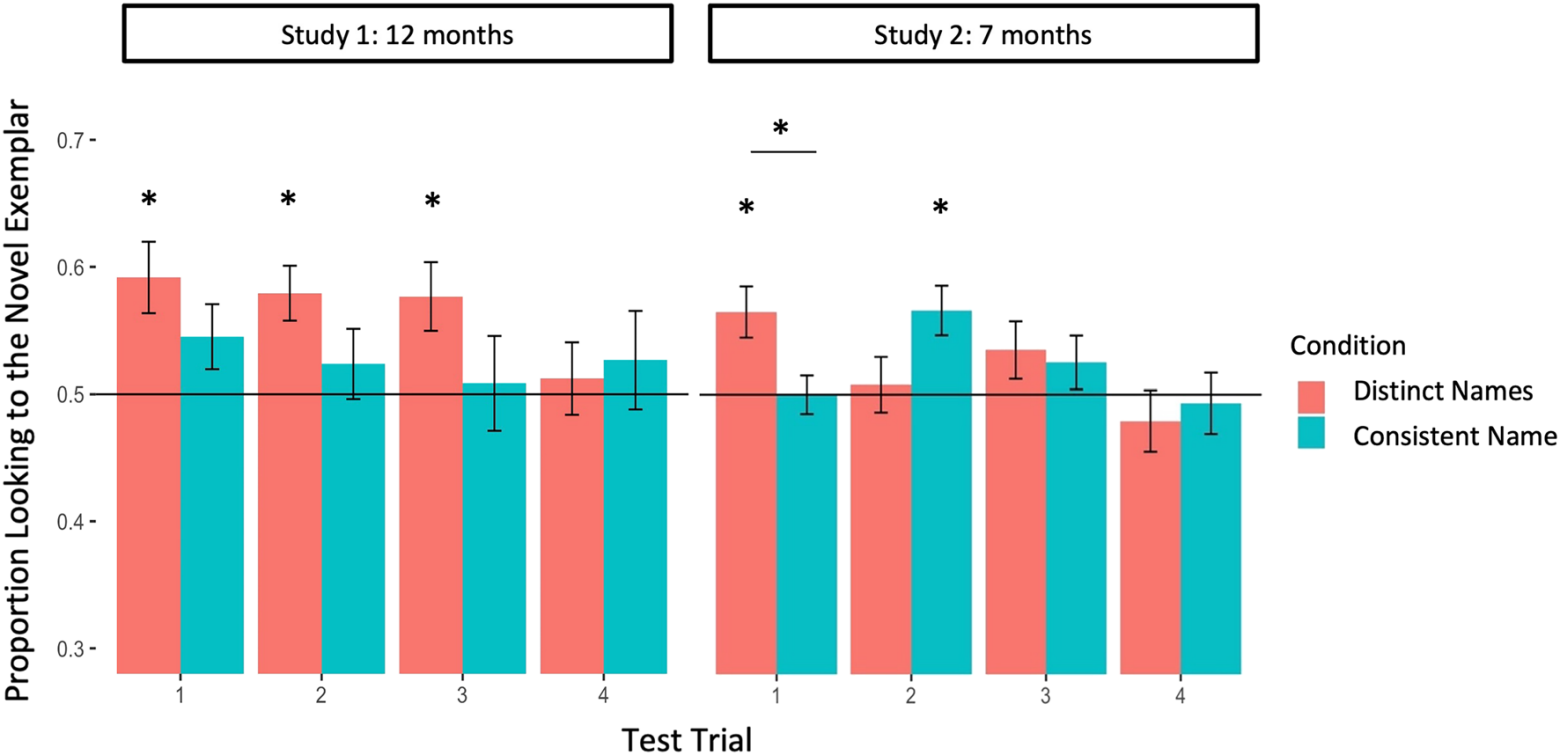
Studies 1 and 2 mean novelty preference scores for Test Trials 1—4 in the Distinct Names and Consistent Name conditions. Error bars represent 1 SEM. Asterisks indicate trials on which performance differs significantly either from a preference of .5 (i.e., equal looking to both objects) or between conditions.

Next, to test the trial-based predictions outlined above, we conducted a series of planned comparisons, comparing infants’ performance on each test trial in each condition against a 50% preference (i.e., equal looking between the two objects), as in the original study. As predicted, infants in the Distinct Names condition favored the novel object on Test Trial 1 (M = 0.59, SD = 0.15; *t*(26) = 3.3, *p* = 0.003, *d* = 0.63) and Test Trial 2 (M = 0.58, SD = 0.11; *t*(25) = 3.7, *p* = 0.001, *d* = 0.72). They also favored the novel object on Test Trial 3 (M = 0.58, SD = 0.14; *t*(26) = 2.8, *p* = 0.009, *d* = 0.54); on Test Trial 4, infants showed no preference (M = 0.51, SD = 0.14, *t*(25) = 0.44, *p* = 0.67). In contrast, infants in the Consistent Name condition did not show a preference for the novel object in any of the four Test Trials, M_Trial1_ = 0.55 (SD = 0.13), M_Trial2_ = 0.52 (0.14), M_Trial3_ = 0.51 (0.19), M_Trial4_ = 0.53 (0.20), *t*s < 1.8, *p*s > 0.05 (see Fig. 2). In this online version of the task, the difference between conditions did not reach significance on any trial, Trial 1: *t*(52) = 1.24, *p* = 0.22, Trial 2: *t*(50) = 1.54, *p* = 0.13, Trial 3: *t*(50) = 1.41, *p* = 0.16, Trial 4: *t*(50) = 0.34, *p* = 0.74.

*Comparing online vs in-lab platforms* Finally, we directly compared results from the online and in-lab platforms^17^. If the online paradigm is viable for assessing the effects of naming on object memory in infants, this analysis should reveal no systematic divergences across platforms. To test this, we constructed a linear mixed effects model including data from both platforms, using random effects of participant and trial-by-participant slopes and fixed effects of Condition, Test Trial, and Platform (i.e., the current online study vs. the in-lab LaTourrette and Waxman^17^ study).

This model revealed no effect of Platform, ﻿β = 0.014, SE = 0.114, *t* = 1.04, *p* = 0.30 and no Platform x Condition interaction, ﻿β = 0.02, SE = 0.027, *p* = 0.45. We also observed no main effect of Condition, ﻿β = 0.018, SE = 0.019, *p* = 0.34, or Test Trial, ﻿β = 0.004, SE = 0.01, *p* = 0.72, but the analysis did reveal a significant Condition x Trial interaction, ﻿β = 0.028, SE = 0.014, *p* = 0.042. To characterize this interaction, we compared performance across conditions on each trial using Welch t-tests. This revealed the predicted pattern of results: better memory for objects in the Distinct Names condition over the Consistent Name condition on both Test Trial 1, t(103) = 2.28, *p* = 0.025, and Test Trial 2, t(94) = 2.23, *p* = 0.028, but not Test Trial 3, t(94) = 1.45, *p* = 0.15, or Test Trial 4, t(94) = 0.92, *p* = 0.36. As a final measure of alignment across platforms, we examined infants’ mean looking preference on each platform for each Test trial in each condition (see Supplementary Table S1). Notably, the mean looking preference in the in-lab study always fell within the 95% confidence intervals from the current, online study, indicating similar trial-by-trial results across platforms.

Thus, the effect sizes in Study 1 were more modest than in the in-lab sample^41, 56^, but the combined analyses revealed that the effect of labeling emerged over time across both the online and in-lab platforms, with labels influencing 12-month-old infants’ memory for the objects introduced on Learning Trials 3 and 4 (and assessed on Test Trials 1 and 2). Together, these analyses provide a strong foundation for assessing the effect of naming on 7-month-old infants in Study 2.

### Study 2: 7 months

Seven-month-old infants participated in the same recognition memory task as in Study 1. Here, we essentially doubled the sample size from Study 1 (to n = 48 per condition) because (a) younger infants’ performance tends to be more variable than older infants’, and (b) effect sizes in Study 1 were more modest than in the in-laboratory sample.

### Predictions

We expected that 7-month-olds, like 12-month-olds, would show better recognition of objects labeled with distinct names rather than consistent names, but that they would reveal this effect only on the first Test trial. This prediction was motivated by evidence that 7-month-olds recall only one object in detail while 12-month-olds successfully recall multiple^25–27, 51^. As in Study 1, we still tested infants’ memory for all exemplars shown during learning.

### Learning phase

Seven-month-olds’ total time looking to the exemplars during Learning was comparable across conditions (M_Distinct_ = 54.29 s, SD = 12.8 s; M_Consistent_ = 52.08 s, SD = 12.35 s; *t*(94) = 0.86, *p* = 0.39). In addition, their total looking time in each condition was comparable to 12-month-old infants’ total looking in each condition in Study 1, *t*s < 1.5, *p*s > 0.2.

### Test phase

We began by constructing a linear mixed effects model predicting preference for the novel object with Condition, Test Trial, and their interaction as sum-coded fixed effects and a random effect of participant and Trial-by-participant random slopes. This model yielded a significant effect of Test Trial (﻿β = − 0.034, SE = 0.016, *p* = 0.04), but no significant effects of Condition (﻿β = − 0.001, SE = 0.016, *p* = 0.9) or the Condition x Test Trial interaction (﻿β = 0.04, SE = 0.033, *p* = 0.3).

We next conducted a series of planned comparisons, comparing performance in each test trial in each condition to a 50% preference. As predicted, on Test Trial 1, 7-month-old infants in the Distinct Names condition looked more to the novel object (M = 0.56, SD = 0.14; *t*(45) = 3.2, *p* = 0.003, *d* = 0.47), but those in the Consistent Name condition showed no preference (M = 0.50, SD = 0.1; *t*(45) = -0.019, *p* = 1). Moreover, performance on Test Trial 1 differed reliably as a function of Condition, *t*(90) = -2.58, *p* = 0.01, *d* = 0.54. This suggests that 7-month-olds, like 12-month-olds, are sensitive to how objects are named, and this has consequences for their recognition memory. While distinct naming helped infants to retain the individuating features of the most recently seen object, consistent naming likely focused infants on category-wide commonalities instead. Test Trial 2, though, yielded an unanticipated difference between conditions, *t*(88) = 2, *p* = 0.05: infants in the Consistent Name condition (M = 0.57, SD = 0.13; *t*(43) = 3.4, *p* = 0.002, *d* = 0.51), but not the Distinct Names condition (M = 0.51, SD = 0.15; *t*(45) = 0.32, *p* = 0.7), favored the novel object. Infants showed no effect of condition on either Test Trial 3 (M_Consistent_ = 0.53, SD = 0.14, M_Distinct_ = 0.53, SD = 0.15, *t*(88) = 0.30, *p* = 0.76), or Test Trial 4 (M_Consistent_ = 0.49, SD = 0.14, M_Distinct_ = 0.48, SD = 0.16, *t*(88) = 0.36, *p* = 0.72) (see Fig. 2).

Next, we compared infants’ performance at 7- and 12-months directly to identify any developmental changes. We constructed a mixed effects model with fixed effects of Age Group (7-month-olds vs. 12-month-olds) and Test Trial, as well as random effects of participant and trial-by-participant slope, for each condition. In the Distinct Names condition, we found a significant effect of Age Group, ﻿β = 0.046, SE = 0.018, *p* = 0.014, indicating that 12-month-olds showed more robust memory for objects than their 7-month-old counterparts. The model also revealed the predicted effect of Test Trial, ﻿β = -0.027, SE = 0.009, *p* = 0.004, indicating that at both 7 and 12 months, infants’ preference for the novel object declined across test trials, in line with the increasing delay between Learning and Test trials. In contrast, performance in the Consistent Name condition did not vary as a function of Age Group, ﻿β = 0.0076, SE = 0.020, *p* = 0.70, or Test Trial, ﻿β = 0.0076, SE = 0.009, *p* = 0.42. Thus, while infants at both ages in the Consistent Name condition generally failed to recognize previously seen category members, infants in the Distinct Names condition recognized as many individuals as permitted by their memory capacity—one for 7-month-olds and three for 12-month-olds.

*A role for visual attention* Finally, we conducted an exploratory analysis, motivated by prior findings that among infants 7 months and younger, greater visual attention during learning was associated with greater success on categorization and recognition tasks^8, 47–50^. To assess whether the same was true for the current task, we identified two groups of 24 infants in each condition – “High Lookers” and “Low Lookers” – determined by whether each infant’s accumulated looking time during Learning was above or below the median. As predicted, the effect of labeling was stronger among the High Lookers. On Test Trial 1, High Lookers in the Distinct Names condition showed a significant preference for the novel object (M = 0.59, SD = 0.12, *t*(22) = 3.6, *p* = 0.002, *d* = 0.75). This preference was significantly greater than that of High Lookers in the Consistent Name condition (M = 0.50, SD = 0.11), *t*(45) = 2.65, *p* = 0.011. In contrast, among the Low Lookers, there was no difference between conditions on Trial 1, *t*(43) = 1.10, *p* = 0.28. On Test Trials 2–4, t-tests comparing performance in the two conditions revealed no significant differences for either High or Low Lookers, *p*s > 0.05 (for these trials’ means and statistical tests, see Supplemental Table S2). Thus, at 7 months, infants who were more attentive during Learning showed the strongest influence of naming on their recognition memory.

## General discussion

These results shed new light on how, and how early, infants become sensitive to the tight coupling between object naming and object representation. Within the first year of life, how an object is named—as either a unique individual or a member of a category—influences how infants encode and remember it. In Study 1, when a series of individual objects all received the same name, 12-month-olds failed to recognize even the most recently seen object from Learning. Yet when each individual object received its own distinct name, infants successfully distinguished them from other, within-category objects, showing recognition for the last three objects seen in Learning. In this online implementation, the difference between conditions at 12 months did not reach significance. Nevertheless, the direction of the results is consistent with prior evidence from more tightly controlled laboratory settings indicating that when the same name is applied consistently to a set of objects, infants increasingly focus on commonalities among the objects, but at the cost of encoding individuating details^17–19^. In contrast, when distinct names are applied to objects, this highlights their unique features—enabling infants to distinguish them from other category members, though likely at the cost of representing these objects’ shared attributes^12, 17, 20^. Study 2 revealed that this link between naming and representation emerges as early as 7 months. This provides support for the proposal that even before infants have acquired a substantial lexicon, they are guided by a principled, conceptually precise link between object naming and representation. This precocious link likely provides a foundation for acquiring some of their first names for object categories (e.g., “cat,” “animal”) and individuals (e.g., “Sammi”).

Our findings reveal both developmental continuity and change. At 7 and 12 months, even a single naming episode affected infants’ encoding of the named object. But infants’ expression of this sensitivity also became more robust from 7 months (when they recognized a single object) to 12 months (when they could recognize three). This is congruent with prior work on the development of infants’ memory for objects^25–27, 51^ and suggests that infants’ increasing attentional and memory capacities can amplify the downstream cognitive consequences of object naming on object encoding and memory.

The current findings also open new avenues for investigation. For example, it will be important in future work to more comprehensively examine the processes underlying infants’ identification of commonalities or distinctions among exemplars, including the extent to which the present results are driven by infants’ attention to the exemplars’ commonalities or to their differences. Another avenue for future investigation will be to clarify the mechanism by which labeling affects 7-month-olds’ encoding of objects, including evaluating 7-month-olds’ unexpectedly above-chance performance on Test Trial 2 in the Consistent Name condition. For instance, if 7-month-olds are slower than 12-month-olds to identify category-wide commonalities among exemplars, then they may still be encoding individuating details on the third learning exposure. Future work might also investigate naming effects with infants acquiring more than one language^52–54^. Previous work suggests bilingual infants may not share their monolingual counterparts’ assumption that each distinct word maps to a distinct object kind ^55^, so it remains an open question whether and when distinct (or consistent) labels influence their encoding of objects. Finally, future work might consider how naming objects with distinct proper nouns, as compared to distinct count nouns, influences objects’ representation. Recent evidence reveals that infants as young as 6 months comprehend the more restricted scope of at least a few proper names^21^. This raises an interesting possibility: as infants become sensitive to surface-level syntactic cues that distinguish proper names from count nouns, then labeling individuals in the Distinct Names condition with proper nouns might yield even more striking effects on infants’ encoding and representation of individual objects.

Altogether, our results show that by 7 months, infants’ representations and memory of individual objects are shaped by how the objects were named: distinct, but not consistent, names facilitated infants’ encoding of each object’s distinguishing features. This suggests that even at the onset of word learning, infants are equipped with an early and powerful link between language and conceptual representations, underpinning their online encoding and memory for objects.

## Methods

### Participants

Participants, recruited from either Lookit or Northwestern University’s Infant and Child Development Center database, received a $5 gift card upon study completion. All caregivers gave informed consent for their child’s participation. All infants were full term (> 37 weeks) and acquiring English (< 30% non-English exposure, per parental report). In Study 1, 64% of families self-identified as White, 4% as Hispanic, 4% as Asian, and 29% as multiracial. In Study 2, 76% of families self-identified as White, 1% as Black, 4% as Hispanic, 6% as Asian, and 13% as multiracial. Studies 1 and 2 included a total of 151 infants.

Study 1 (12-month-olds) included 28 infants in the Distinct Names condition (M_age_ = 11;30, range: 11;16–12;19, 12 female, 16 male) and 27 in the Consistent Name condition (M_age_ = 11;27, range: 11;16–12;11, 14 female, 13 male). This sample size (designed to include at least 24 datapoints per test trial) provided approximately 85% power, based on the effect size of d = 0.88 reported for the first test trial in LaTourrette and Waxman^17^. We focused on this first trial effect, rather than an effect across all trials, as we expected the effect of condition to be present on each of the first two test trials but not subsequent trials, as in the original study.

Study 2 (7-month-olds) included 48 infants in the Distinct Names condition (M_age_ = 7;22, range: 7;0–8;15, 21 female, 27 male) and 48 in the Consistent Name condition (M_age_ = 7;21, range: 6;30–8;18, 23 female, 25 male). A sample size of 48 infants per condition (Study 2) provided 99% power, based on LaTourrette and Waxman’s^17^ effect size of d = 0.88 and alpha = 0.05.

An additional 49 families who consented were excluded from analysis, either because the infant failed to attend to the objects for at least 25% of the Learning Phase or 25% of at least three different Test trials (Study 1: 4, Study 2: 8; see Coding and Analysis below), because of platform-specific problems with stimulus presentation (Study 1: 15, Study 2: 18), or because the quality of infants’ video recordings was too poor for coding (Study 2: 4).

All methods conform to the standards outlined in the Declaration of Helsinki and were approved by the Northwestern University Institutional Review Board.

## Materials

All stimuli were identical to LaTourrette and Waxman^17^. Visual materials were images of eight stuffed animals (Fig. 1). Auditory materials were labeling phrases (~ 3800 ms duration) recorded in a soundproof booth by a female, native English speaker and featuring two utterances of the novel label (e.g., “Look at the *arg*! Do you see the *arg*?”).

### Procedure

All families participated from their homes, using their own computer and webcam, via the Lookit platform ^41^. After consenting, caregivers were instructed to hold their infants over their shoulders, turning their own backs to the screen so that they could not influence their infant’s attention to the images. Otherwise, the procedure was identical to LaTourrette and Waxman^17^. Infants participated in two phases: Learning and Test (Fig. 1).

*Learning phase* Infants were randomly assigned to either the Distinct Names or Consistent Name condition, differing only in how the exemplars presented in the Learning Phase were named. To begin, an attention-getter (a colorful, spinning wheel) appeared alternately on each side of the screen to attract infants’ attention to both sides. Next, infants viewed images of four different animals, presented one at a time for 20 s each, on alternating sides of the screen. In the Distinct Names condition, a different novel name was paired with each object. In the Consistent Name condition, the same novel name was paired with all four objects. The side on which the first image appeared and the label(s) paired with the objects were randomly determined.

*Test phase* To begin each trial, the attention-grabber appeared at the center of the screen (5 s). Next, a pair of objects appeared side-by-side in silence for 20 s. Each pair included one animal that infants had seen previously during Learning (familiar object) and one they had not seen (novel object). As in LaTourrette and Waxman^17^, the familiar objects were presented in the reverse order from the Learning phase. That is, the object presented on Learning Trial 4 was presented on Test Trial 1.

## Coding and analysis

Infants’ visual attention throughout was coded manually by a trained research assistant offline ^57^. Infant gaze was coded as center, right, left, or off screen. Intercoder reliability, computed by a second trained coder for 21 videos in Study 1 and 17 videos in Study 2, was excellent (Study 1: Pearson’s *r* = 0.96, *p* < 0.001; Study 2: Pearson’s *r* = 0.95, *p* < 0.001).

For Learning trials, we calculated infants’ total looking time to the objects on all four trials. For Test trials, we used the first 10 s of accumulated looking in each test trial to calculate a novelty preference score, defined as time looking at the novel test object/time looking at the novel and familiar test objects combined^8, 17, 36^. These preference scores, expressed as proportions, were arcsine-square-root transformed for linear mixed effects models in lme4^58, 59^, with fixed effects for Condition (sum-coded), Test Trial (coded as a linear polynomial contrast), and the Condition x Test Trial interaction, as well as a random effect of Participant. To assess significance, we used the Satterthwaite approximation for degrees of freedom to minimize type I error^60^, as implemented in the lmerTest package^61^ in R^62^.

We included in analysis only those test trials in which the infant had looked at that object for at least 5 s during Learning, looked to both objects at Test, and devoted at least 5 s of looking (25% of the trial) to the objects during Test.

### Supplementary Information

Supplementary Tables.

## Data Availability

The data that support the findings of this study are openly available at https://osf.io/zfdev/.
