# Supplementary material for: A principled link between object naming and representation is available to infants by seven months of age
Source: Sci Rep. 2023 Aug 31;13:14328. doi: 10.1038/s41598-023-41538-y (PMC10471589; doi:10.1038/s41598-023-41538-y)
Supplement: Supplementary file 1 — Supplementary Tables. [file 41598_2023_41538_MOESM1_ESM.pdf]

Supplementary Table 1

Mean Novelty Preferences from LaTourrette & Waxman (2020), Study 1 (12 mo), and Study 2 (7 mo)

Study	Age Group	Condition	Test Trial	Mean Novelty Preference (SE)	95% CI
**LaTourrette & Waxman 2020 (in lab)**	12-months	Consistent	1	.517 (.013)	[0.49,0.54]
**LaTourrette & Waxman 2020 (in lab)**	12-months	Consistent	2	.498 (.020)	[0.46,0.54]
**LaTourrette & Waxman 2020 (in lab)**	12-months	Consistent	3	.541 (.019)	[0.5,0.58]
**LaTourrette & Waxman 2020 (in lab)**	12-months	Consistent	4	.546 (.027)	[0.49,0.6]
**LaTourrette & Waxman 2020 (in lab)**	12-months	Distinct	1	.573 (.017)	[0.54,0.61]
**LaTourrette & Waxman 2020 (in lab)**	12-months	Distinct	2	.541 (.014)	[0.51,0.57]
**LaTourrette & Waxman 2020 (in lab)**	12-months	Distinct	3	.555 (.022)	[0.51,0.60]
**LaTourrette & Waxman 2020 (in lab)**	12-months	Distinct	4	.504 (.026)	[0.45,0.55]
**Study 1 (online)**	12-months	Consistent	1	.545 (.026)	[0.49,0.60]
**Study 1 (online)**	12-months	Consistent	2	.524 (.027)	[0.47,0.58]
**Study 1 (online)**	12-months	Consistent	3	.508 (.036)	[0.44,0.58]
**Study 1 (online)**	12-months	Consistent	4	.527 (.038)	[0.45,0.60]
**Study 1 (online)**	12-months	Distinct	1	.592 (.028)	[0.54,0.65]
**Study 1 (online)**	12-months	Distinct	2	.579 (.021)	[0.54,0.62]
**Study 1 (online)**	12-months	Distinct	3	.577 (.027)	[0.52,0.63]
**Study 1 (online)**	12-months	Distinct	4	.512 (.027)	[0.46,0.57]
**Study 2 (online)**	7-months	Consistent	1	.500 (.015)	[0.47,0.53]
**Study 2 (online)**	7-months	Consistent	2	.566 (.019)	[0.53,0.60]
**Study 2 (online)**	7-months	Consistent	3	.525 (.020)	[0.49,0.56]
**Study 2 (online)**	7-months	Consistent	4	.493 (.024)	[0.45,0.54]
**Study 2 (online)**	7-months	Distinct	1	.565 (.020)	[0.53,0.60]
**Study 2 (online)**	7-months	Distinct	2	.508 (.021)	[0.47,0.55]
**Study 2 (online)**	7-months	Distinct	3	.535 (.022)	[0.49,0.58]
**Study 2 (online)**	7-months	Distinct	4	.479 (.023)	[0.43,0.52]

Supplementary Table 2

Mean Novelty Preferences (NP) for Low Lookers vs. High Lookers in Study 2

Group	Test Trial	Consistent Name Mean NP (SD)	Distinct Names Mean NP (SD)	*t* statistic	*p* value
**High Lookers**	1	.50 (.11)	.59 (.12)	2.65	.011
**High Lookers**	2	.56 (.11)	.52 (.16)	.95	.35
**High Lookers**	3	.51 (.11)	.54 (.17)	.69	.50
**High Lookers**	4	.51 (.13)	.48 (.18)	.72	.48
**Low Lookers**	1	.50 (.09)	.54 (.15)	1.10	.28
**Low Lookers**	2	.58 (.15)	.50 (.13)	1.88	.067
**Low Lookers**	3	.54 (.16)	.53 (.14)	.24	.81
**Low Lookers**	4	.47 (.19)	.48 (.14)	.14	.89

Note. Low Lookers were defined as those infants who showed below-median attention to the screen during the Learning phase; High Lookers were those infants who showed above-median attention to the screen during the Learning phase.
